# Asphalt-Cement Concretes with Reclaimed Asphalt Pavement and Rubber Powder from Recycled Tire

**DOI:** 10.3390/ma14092412

**Published:** 2021-05-06

**Authors:** Jerzy Kukiełka, Wojciech Bańkowski, Krzysztof Mirski

**Affiliations:** 1 Department of Roads and Bridges, Lublin University of Technology, Nadbystrzycka 40, 20-816 Lublin, Poland; 2 Road and Bridge Research Institute, ul. Instytutowa 1, 03-302 Warsaw, Poland; wbankowski@ibdim.edu.pl (W.B.); kmirski@ibdim.edu.pl (K.M.)

**Keywords:** reclaimed asphalt pavement, asphalt-cement concrete, cement mortar, rubber powder

## Abstract

The goal of the work was to describe properties of asphalt-cement concrete (ACC) with reclaimed asphalt pavement (RAP), Portland cement, sand, and rubber powder (RP), as a material to base courses of road pavements. The mixtures were designed with the RAP in the amount of 75, 80, and 85% (m/m) and chosen cement-sand-rubber (CSR) mortar. Three CSR mortars were composed with cement CEM 42.5 R in the amount 29% (m/m); washed sand 0/2 mm in the amount 29, 35, or 41%; rubber powder of granulation 0/1 mm in the amount of 18, 24, or 29% (m/m); and water in the amount 12% fulfilled w/c = 0.4. The optimum moisture content of the selected ACC with CSR mortar determined in the modified Proctor compaction test was approximately 6% and maximum dry density 2.000 g/cm^3^. Laboratory tests of indirect tensile strength, stiffness modulus (IT-CY and 4PB-PR), water resistance, fatigue life, and complex modulus (E*) at different temperatures were conducted and analyzed. The test results are presented, among others, in the form: the isotherm of complex modulus, Black curve, the master curve, and the Cole-Cole plot.

## 1. Introduction

### 1.1. Reclaimed Asphalt Pavement

Reclaimed asphalt pavement (RAP) is mostly used to produce “hot” or “warm” bituminous mixtures and to a lesser extent for “cold” technology. The use of RAP as an aggregate substitute in road asphalt concrete (HMA and WMA) in the USA was initially allowed from 10 to 20% [1]. Unfractionated RAP has been used in Texas as a replacement for aggregate up to 30% [2]. Data provided by 16 countries to the European Asphalt Paving Association (EAPA) in early 2020 on the reuse and recycling of old asphalt pavements showed that of the 49.5 million tons of recovered RAP available in these countries, 76% was used to produce new bituminous mixtures and 20% was recycled in unbound road layers and other engineering applications. This paper is focused on “cold” recycling.

### 1.2. Mineral-Cement-Emulsion Mixtures

The properties of mixtures containing RAP with virgin aggregate and cement-emulsion mortar are known for practical use as mineral-cement-emulsion mixtures (MCEM). In Poland, first requirements were published in 1999 by IBDiM Issue 61 [3], since then many years of laboratory and field research [4,5,6,7,8,9,10,11] also FWD measurements of pavement after 13 years of using [12] have been made. The disadvantages of MCEM base courses in Poland include mainly: transverse cracks, accelerated degradation in the weakest places of the layer where fatigue damage has reached the value of D = 50%, the commonly used “in situ” method that does not ensure uniformity of the layer made [12]. Publications [13,14] include test results of IT-CY stiffness modulus, complex modulus (E^*^), phase angle, master curves, and low-temperature resistance. Cylindric samples of MCEM containing 5% (m/m) cement, 3% (m/m) asphalt emulsion, 50% (m/m) RAP, and 42% (m/m) virgin aggregate were tested for fatigue life by the compression and tension method at 10 °C. There was found the decrease in stresses corresponding to constant tensile strains ε_t_ = 80 µm/m meeting the durability criterion E/E_0_ = 0.5 after 10^6^ cycle load and, exceeding which, to ε_t_ = 196 µm/m, the decrease was E/E_0_ = 0.2. The report on the analysis of pavements with base course made of MCEM made in 2006 and the first stage in 2011 and the second stage in 2012 of research on this technology allowed to develop a new design procedure in 2014 [15] and typical pavement structures given in the catalogue of typical pavement structures [16].

### 1.3. Asphalt-Cement Concrete

Asphalt-cement concrete (ACC) as a composition of RAP and cement-sand mortar in the amount from 15 to 20% (m/m) was used locally as the base course. Laboratory tests of asphalt-cement concretes, initiated in 1995 at the Lublin University of Technology, were the basis for making in 1997 and 1998 of several intersections and bus stops with this technology [17]. The RAP content is from 70 to 85% (m/m) in ACC, related to the cement-sand mortar, containing most often cement 52.5 in the amount of 7.5% (m/m), sand 0/2 mm in the amount of 7.5% (m/m), and water w/c = 0.6. ACC mix was characterized by indirect tensile strength ITS = 1.07 ÷ 1.47 MPa at the temperature of 20 °C and stiffness modulus of 23,773 MPa at the temperature of 0 °C to 5742 MPa at the temperature of 40 °C. Numerous examples of ACC studies are given in publication [18].

The 0/2 mm fraction content in RAP in the range of 10% ÷ 35% has an impact on the amount of cement-sand (CS) mortar added. It was assumed that the cement sand mortar C:S = 1:1 supplemented with small RAP aggregates plays a role as a binder in an amount of 15% (m/m) to mixture.

On the ACC base course, binder and wearing course of asphalt concrete also SMA can be used. The axial compressive strength of ACC samples taken from the counter-rotating concrete mixer on site was 5.0 ÷ 6.0 MPa after 28 days of hardening and insignificantly less after seven days due to the use of 52.5 cement. The thermal expansion coefficient is, for example, relatively low in the case of dolomite and granite aggregates and high in the case of quartzite sands. A low humidity of the hardened cement paste is a favorable factor. Infrequent transverse cracks have not been the subject of detailed studies of the pavement with ACC. It is possible that cracks in the subbase under the ACC base course are transferred as reflected cracks on the road surface. Vibrations caused by the movement of heavy vehicles, especially at low ambient temperatures, can also cause ACC cracks. Based on the Benkelman beam and the measurement of the rut depth, favorable results were found when ACC was made on an old paving surface made of clinker or semi-rigid subbase. The reconstruction of intersections with ACC base courses has been in use for over 20 years [18].

The bitumen coating the grains and particles of RAP and the cement mortar in ACC ensure its semi-brittle properties as well as viscoelastic deformations. The influence of ambient temperature on ACC depends mainly on the characteristics of the matrix, especially the type of binder and its amount. The cement-sand mortar in ACC ensures its elastic properties and contributes to its brittle cracking.

Base courses with ACC, the characteristics of the RAP, the results of laboratory tests, and practical experiments are summarized in the co-author’s monograph in 2013 [19].

The publication of 2007 [20] contains the results of ACC samples tests with mortar with cement 42.5. Among others, the following were conducted: stiffness modulus IT-CY, creep modulus under quasi-static load as well as the influence of 2/4 mm grit addition to ACC on mechanical properties. The stiffness modulus of the ACC samples was 11,163 MPa at the temperature of −2 °C and 3717 MPa at the temperature of 40 °C (the range of strain was assumed to be ε_t_ = 20 ÷ 55 µm/m). Based on the obtained test results under quasi-static load, the parameters according to the Burgers, Calvin-Voigt, Hook, and Newton models were calculated and a preliminary fatigue life assessment was carried out. The parameters of the Burgers model of ACC samples were calculated under a load of 0.6 MPa at 20, 40, and 60 °C temperatures. The influence of temperature on plastic deformation is noticeable. ACC mixes with cement-sand mortar, tested at a load of 0.35 MPa, were characterized by strains, which, at a load of 0.6 MPa, occur in mixes with cement-sand-gravel mortar. The addition of 2/4 mm grit to the cement-sand mortar increases the indirect tensile strength and therefore increases the components of the elastic part in the Burgers model. The strength increase can be doubled if the content of size 2/4 mm aggregate is circa 8% of the ACC. The addition of aggregate can also help to reduce the amount and type of cement in ACC. The quasi-static loads have been used in ACC research also in correlation with their practical application in bus bays and intersections at planned reconstruction sites.

### 1.4. Asphalt-Portland Cement Concrete Composite in HMA

Special mortar to composite with HMA has been used in France. To the “hot” HMA mix with AC-20 binder, dolomite aggregate pre-compacted in a Marshall press (with a void content of 25% ÷ 35%) cement, fly ash, sand, and water with Prosalvia resin (PL7) were added [21]. Preparation of the composite (asphalt-Portland cement concrete composite, APCCC) consisted in wrapping with tape the samples previously filled with liquid mortar and compacting them on a vibrating table. Vibration should not segregate components and it was assumed that proper filling of air voids takes place when there are no air bubbles on the sample surface. The average bulk density of the PCC samples was 2310 kg/m^3^, the filling with mortar was 93%, and the air voids in the sample was 2.2%.

Fine-, medium-, and coarse-grained dolomite aggregate and binder in the amount of 3.7, 3.9, 4.3, and 4.5% were used for the tests, and the optimal content was 4.05% of the aggregate mass according to the empirical formula.

Mortars were made according to the composition, in which Portland cement constituted 38.5%, fly ash 19.2%, sand 12.7%, water 26.8%, and Prosalvia resin 2.8%. The resin increases strength and facilitates penetration of the mortar into the aggregate coated with the binder. Based on laboratory tests, it was recommended to use coarse aggregate. According to the work summary, all tested properties showed a significant improvement compared to typical “hot” asphalt pavement mixes in the first days of ACC hardening the pavement should be protected from heavy vehicle loads due to insufficient indirect tensile strength, which increases rapidly during the first seven days.

### 1.5. Use of RAP in Cement Concrete

RAP is also used in cement concrete (RAP-PCC) [22,23]. The authors of the research [23] at the Institute of Transport in Texas in 2017 carried out extensive research on RAP containing bitumen from 4 to 6.19% from unfractionated RAP for further tests segregated to coarse-grained fractions screened on a 9.5 mm sieve, medium-grained passing through a 9.5 mm sieve, and remaining on a 2.36 mm sieve and fine grain passing through a 2.36 mm sieve.

The initial work on the evaluation of RAP as substitute of aggregate depending on the amount of bitumen was supplemented with the conclusion that the rate of reduction of the bending strength with the increase of the RAP addition is lower compared to the compressive strength.

The guidelines and recommendations for the design of RAP-PCC developed by the authors include [23]:

- preparation of mixtures containing 20, 30, and 40% of RAP;
- tests after 28 days of curing for indirect tensile strength (exceptionally for compression);
- determination of bitumen volume content in RAP-PCC;
- bending tensile strength should be ≥3.93 MPa, and compressive strength ≥ 26.6 MPa.

Cracking properties of RAP-PCC samples with and without a notch were comparable to the control PCC samples [24,25]. The bitumen coating the aggregate in RAP helps the propagation and elongation of the crack path compared to the brittle cracks in PCC [26,27].

The use of RAP in PCC in road pavements was the subject of research in France at the beginning of the 21st century. Cement concretes containing 330 kg/m^3^ and 220 kg/m^3^ of cement were tested as comparative concretes to RAP-PCC, with a grain size of 0/20 mm and a bitumen content of 5.4% (m/m), as an aggregate substitute in the amount of 15, 30, 60, and 100% [28].

The research [29] may be useful in the case of using RAP also for concretes with continuous reinforcement [30] and for “white topping” surfaces [31].

### 1.6. Use of Rubber Granulated Materials

The recycling of car tires and the further use of products from them were the subject of the US laws in the 20th century [32] and extensive research work in most countries struggling with landfills of used car tires. Practical applications, especially in “hot” mixes, in the 21st century have mechanically grounded rubber at ambient temperature, as it allows to obtain about twice the specific surface area than that obtained by the cryogenic method [33].

Crumb rubber is most often used as a binder component in the “wet process” and as an additive in the “dry process” for “hot” bituminous mixtures or to produce mineral mixtures using the “cold” technology with cement [34,35,36,37,38,39]. There are numerous results of studies on the influence of separately added RAP and tire recycled rubber on the properties of the mixtures [40,41,42,43].

The adhesion effect between the cement matrix and the crumb rubber is particularly important when used in Portland cement concerts (PCC). Rubber waste in PCC most often replaced coarse aggregates. The most frequently observed effect was the reduction of compression strength and indirect tensile strength [44]. The research work [45] on PCC with rubber crumbs found a beneficial effect on bending strength and impact load. The greatest reduction in compressive strength by 45% was found in PCC with the addition of 15% rubber crumbs, while in the case of indirect tensile strength by 25% [41,46]. The publications state that this is mainly due to the hydrophobic characteristics of rubber and poor interfacial relationships. The “hole” effect in PCC increases with increasing dimensions of granules (from 1 to 10 mm) or crumb rubber [46]. The increase of ductility and reduction of brittle fractures in PCC with rubber crumbs was used for making transition plates before bridges, for example [47]. The rubber modification in PCC may reduce water and frost resistance. There are fragmentary tests of frost resistance of PCC with rubber crumbs, performed by the peeling mass method of the upper surface of the sample (the method of the Swedish standard SS 137244, which is a modification of ASTM C 672), despite the good assessment are insufficient, according to the authors of the research [48].

Rubber particles block the diffusion of water and inhibit the hydration of cement [49]. It may be beneficial to increase the stiffness of the rubber particles by coating them with cement-based materials [50]. Chemical treatment of rubber particles, e.g., with NaOH solution started in the 20th century, did not bring significant effects [51].

Structural damage of PCC with the addition of a relatively large amount (about 4% m/m) of rubber powder caused by frost should be the subject of separate studies, e.g., in the countries of Central and Northern Europe. The choice of a research method is of fundamental importance. The methods of ASTM C 666 A and B, ASTM C 671 and C 672, SS 137244 are known from the experience, and the test described by RILEM CDF and Cube, published in the last decade of the twentieth century, contain 16–18 parameters characterizing their range [52].

A comprehensive review of rubber waste tests [53] (84 references) and their use states that the addition of crumb rubber may have a slightly negative effect on the resistance to freezing [48].

The tests of cement and cement-asphalt matrices with the addition of rubber powder [54] were used to accept the compositions of mortars for ACC and to interpret the results of the research conducted by the authors of this work.

The initiated tests of fatigue life of the mineral-cement-emulsion mixture with the addition of 0/2 mm rubber powder from recycling of car tires were carried out based on the experiments concerning asphalt concretes at the beginning of the 21st century [55].

### 1.7. Fatigue Life of Materials

Fatigue cracks can be generated at the bottom of the asphalt layers and propagate upwards. The beginning of cracks in the wearing course was considered in the AASHTO 2002 pavement design method [56]. The theoretical scope of the fatigue crack growth enables its division into three zones: initiation, propagation, and material failure (fracture) [57]. The analysis of changes in the stiffness modulus diagram under the influence of load cycles using the axial compression-tension method and the beam bending method was the subject of the work [58] published in 2002. In the case of tests using the controlled strain method, the fatigue life increases with the increase of the test temperature, the decrease in durability occurs with an increase in the stiffness modulus of the tested mixture, the effect of rest (relaxation) is small, a decrease in fatigue life is observed with an increase in the load frequency [59,60].

The accumulation of fatigue damage, called the fatigue damage (D), is subject to the Miner’s hypothesis [61], and the parameters of Whöler’s equations define the slope of the fatigue life depending on the strain. The results of fatigue tests at various temperatures enable the determination of complex equations of the fatigue characteristics. Schemes of homogeneous fatigue methods are included in RILEM Report 17. The cyclic bending of the beams was adopted in the SHRP method [62], which is also useful in later studies of mixtures with modified binders [63].

The analysis of the fatigue phenomenon should only concern phase II, starting after about 50,000 cycles. Sample degradation in phase III is caused by the heterogeneity of the material with the propagating and joining microcracks.

## 2. Constituent Materials for the Production of ACC with Rubber Powder

The mortars were made of class I Portland cement, Górażdże Cement S.A., Chorula, Poland, characterized by compressive strength of 42.5 with high early strength “R” as per EN 197-1 [64], washed sand 0/2 mm, and rubber powder with grain size 0/1 mm according to the manufacturer’s data. RAP with bitumen content in mixture B = 5.3% ÷ 7.5% (m/m) was used for the preparation of ACC samples. The grading of the materials is presented in Table 1.

RAP was collected from the material storage yard of KPRD Limited Liability Company in Lublin, Poland, rubber powder was provided by the producer Orzeł S.A. from Poniatowa, Poland.

## 3. Types and Methods of Sample Preparation

The compositions of cement-sand-rubber CSR mortars is presented on Table 2.

The maximum bulk density was obtained in the standard Proctor test I [65] (small mold, 25 blows of the light rammer on each of 3 equal layers) on CSR mortars “A”, “B”, and “C”, respectively ρ_Admax_ = 1.788 g/cm^3^, ρ_Bdmax_ = 1.678 g/cm^3^, ρ_Cdmax_ = 1.583 g/cm^3^.

CSR mortars samples Ø100 × 63.5 mm were made by compacting in a Marshall compactor by 30 strokes per side to obtain a density corresponding to the standard Proctor test. From each composition, 4 samples Ø100 × 63.5 mm were made for ITS and IT-CY tests.

**Figure 1:**
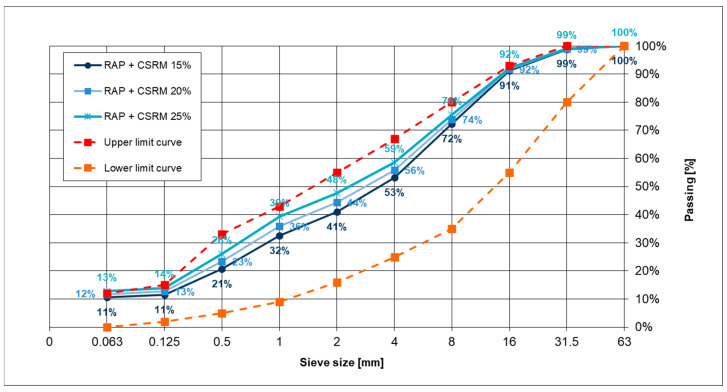
Grading curves of a mineral mix with the addition of “B” cement-sand-rubber mortar applied in the amount of 15, 20, and 25% (m/m).

The particle size distribution curves of ACC mixtures with “B” CSR mortar are shown in Figure 1.

ACC composition was with selected “B” CSR mortar added in amount of 15, 20, 25% (m/m) and RAP in amount of 85, 80, 75% (m/m), respectively, to the mixture. All samples were compacted at room temperature circa 20 °C as in “cold” technology.

The asphalt-cement concrete with rubber powder (ACCR) samples with “B” mortar with dimensions of Ø100 × 63.5 mm was made by compacting in a Marshall compactor with 50 blows per side to obtain a density corresponding to the modified Proctor test IV [65] (large mold, 55 blows of the heavy rammer on each of 5 equal layers).

The ACCR plates with dimensions of 300 mm × 40 mm × 70 mm with the “B” CSR mortar in the amount of 15, 20, and 25% were made in a plate compactor until the maximum bulk density of 2.000 g/cm^3^ determined in the modified Proctor test was obtained.

Beams for stiffness module and fatigue tests with dimensions of 380 mm × 63 mm × 50 mm were made by cutting them out of the plate, compacted, and the compaction index corresponded to the requirements of ≥98%.

The ACC maximum bulk density with 20 and 25% “B” CSR mortar was ρ_20dmax_ = 1.975 g/cm^3^ and ρ_25dmax_ = 1.955 g/cm^3^, respectively. Air voids obtained on samples was 14.0% < V < 16.0%.

**Figure 2:**
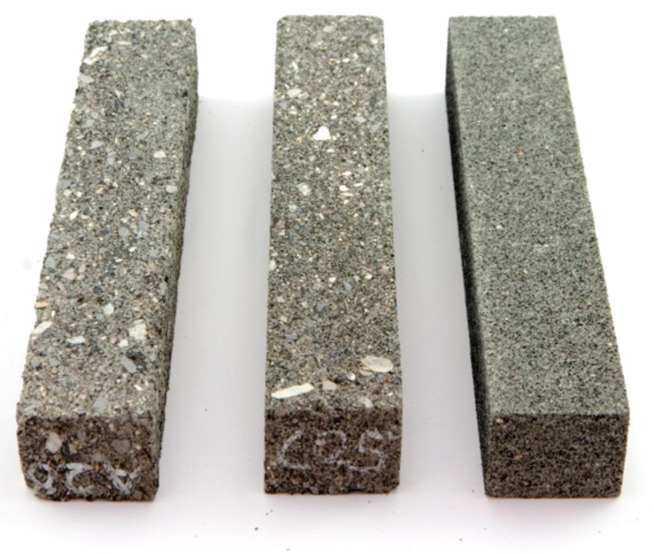
Beams 380 mm × 63 mm × 50 mm of ACC with “B” mortar, and “B” CSR mortar alone for testing the stiffness modulus using 4PB-PR method.

Samples of CSR mortar and ACCR are shown in the Figure 2.

## 4. Testing Plan

Tests of indirect tensile strength ITS, stiffness modulus by the IT-CY, and 4PB-PR methods were carried out at a wide range of temperatures to determine the influence on the mechanical properties of ACC with rubber powder. Before testing, all samples were stored for at least 4 hours in the air-conditioning chamber to obtain the required temperature.

### 4.1. Indirect Tensile Strength

The indirect tensile strength tests were carried out in accordance with requirements of the PN-EN 12697-23 [66] at the temperature of 5 °C in accordance with the recommendations of the technical guidelines [15].

### 4.2. Water Resistance

The water resistance tests were carried out in accordance with the technical guidelines [15]; however, due to the different time needed to obtain full saturation of the matrices, the method of weighing the sample after each soaking day until a constant mass was obtained, was used.

Water resistance as the remaining indirect tensile strength after storing the samples in water was calculated according to the formula [15]:

(1) $$ITSR=100\;\times \;\frac{ITS_{s}}{ITS_{d}}$$

where:

*ITSR*—index of indirect tensile strength after soaking the samples with water [%]
*ITSs*—average indirect tensile strength of water-soaked samples [MPa], calculated using the formula:
  (2) $${ITS}_{s}=\frac{2\times P_{s}}{\pi \times D\times h}$$
*ITS_d_*—average indirect tensile strength of dry specimens [MPa], calculated using the formula:
  (3) $${ITS}_{d}=\frac{2\times P_{d}}{\pi \times D\times h}$$
*P_s_*, *P_m_*—maximum value of the compressive force [N],
*D*—sample diameter rounded to 0.1 mm,
*h*—specimen height rounded to 0.1 mm.

### 4.3. Stiffness Modules

The stiffness modulus was made on Marshalls samples in accordance with the requirements of the EN 12697-26 standard [67], Annex C, by measuring vertical and horizontal displacements at mid-height of the specimen under controlled load.

The stiffness modulus on beam was carried out using the four-point bending method in accordance with PN-EN 12697 26 standard.

### 4.4. Fatigue Life

Fatigue life was tested using the four-point bending method on prismatic specimen (4 PB-PR) in accordance with the EN 12697-24 standard [68] after 28 days of curing the plates.

Standard conditions were adopted: the temperature of 10 °C and load frequency of 10 Hz. The mode of controlled strains with sinusoidal signal was used. The test was performed in a servo-hydraulic press manufactured by MTS (Material Test System) at different values of the strain amplitude to determine the fatigue characteristics, described with the Wöhler equation:

(4) $$N=A\times \varepsilon^{b}$$

where:

*N*—fatigue life according,
*ε*—strain amplitude in fatigue test [mm/m],
*A*, *b*—linear regression parameters.

The fatigue life test was carried out assuming the classical criterion of 50% decrease in the stiffness modulus.

### 4.5. Complex Stiffness Modulus and Master Curves

Testing of the complex stiffness modulus was carried out in accordance with EN 12697-26 [67] by a four-point bending beam (4PB) method. The test results were the stiffness modulus E^*^ and the phase angle θ. The phase angle or its tangent is an indication of the dominance of viscous or elastic properties in the material: the lower its value, the more elastic the material is. The complex stiffness modulus E^*^ can be represented as a complex number, which consists of the real (elastic) part E′ and the imaginary (viscous) E″. Temperatures −2, 10, 23, 40 °C and frequencies from 0.5 to 15 Hz were adopted in tests. Master curves were described by the sigmoidal function, which use the principle of the so-called time-temperature superposition described by Williams-Landel-Ferry [69]. The master curve shows the test results brought to reference temperature of 13 °C as a function of reduced frequency [70]. The development of the master curves of the stiffness modulus E and the phase angle θ used the equation (Equation (5)) proposed by Francken and Vanelstraete [71]:

(5) $$\log(a_{T})=\frac{\delta H}{R}(\frac{1}{T}-\frac{1}{T_{S}})$$

where:

δH—material-specific activation energy (estimated in optimization for each mixture),
*R*—universal gas constant, 8.31 J/mol/K,
*T*—temperature, °K,
*Ts*—reference temperature, °K.

The master curve of the complex modulus norm has been approximated by the following function [72]:

(6) $$E\left(f_{r}\right)=E_{0}+\left(E_{\infty }-E_{0}\right)\frac{f_{r}^{c}}{f_{r}^{c}+d}$$

where *f_r_* means reduced frequency, and *E_0_*, *E_∞_*, *c*, and *d* are parameters. These parameters were set by solving the issue of minimization of mapping error compared to experimental test results. Non-linear optimization methods with constraints (*E*_0_ > 0) were used, determining in the first step the parameters of the function (6) and the activation energy value from the Equation (5). In the second step, parameters were determined in the function describing the master curve of the phase angle in the form of:

(7) $$\theta \left(f_{r}\right)=\theta_{0}\frac{a\;f_{r}^{\alpha }+1}{b\;f_{r}^{\beta }+1}$$

I.e., parameters *a*, *b*, θ_0_, α and β. In this case, the non-linear optimization algorithm with constraints (β > α) was used once again. It is worth noting that in the second step, the activation energy value was assumed as given value.

## 5. CSR Mortars Test Results and Analysis

**Figure 3:**
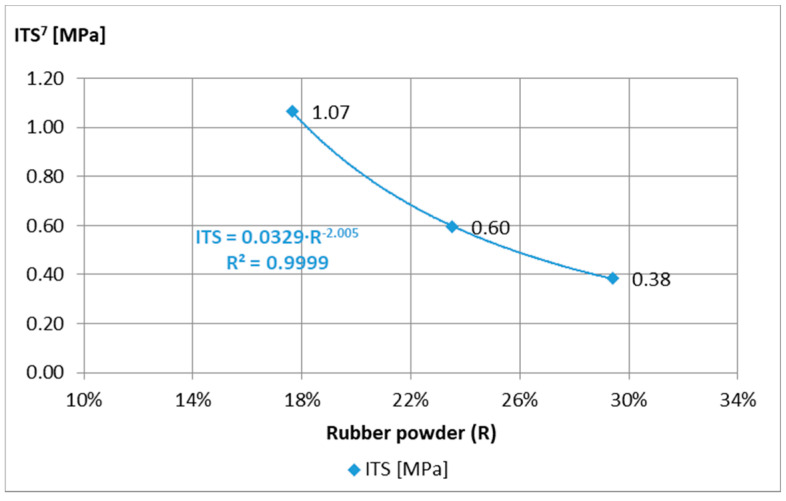
Indirect tensile strength ITS of mortar samples Ø100 mm × 63.5 mm depending on the amount of rubber powder, tested at the temperature of 5 °C, after 7 days of curing.

**Figure 4:**
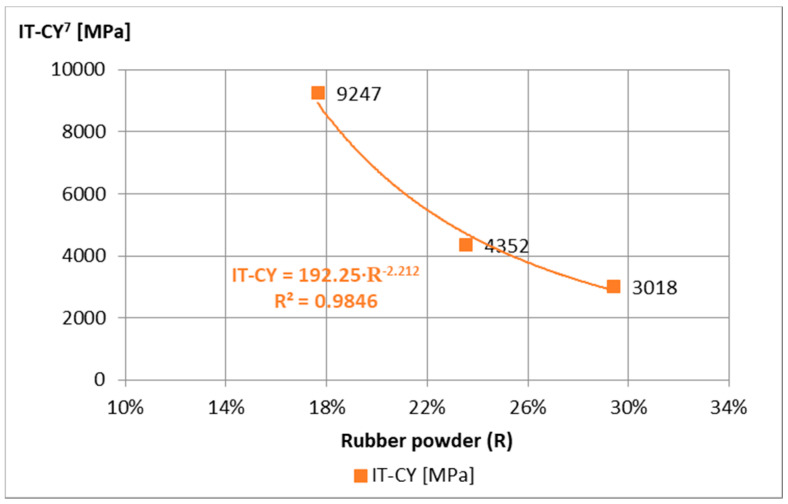
IT-CY stiffness modulus of mortar samples Ø100 mm × 63.5 mm depending on the amount of rubber powder, at 5 °C, after 7 days of curing.

For the analysis of the obtained test results of mortar samples “A”, “B”, and “C”, the requirements of the technical guidelines [15] were adopted, on the basis of which the 7-day strength should be 0.4 MPa < ITS^7^ < 0.8 MPa for traffic category TC 1–2 and 0.6 MPa < ITS^7^ < 1.0 MPa to TC 3–4, and the stiffness modulus tested after 28 days are 1500 MPa < IT-CY^28^ < 5000 MPa and 2000 MPa < IT-CY^28^ < 7000 MPa, respectively. The minimum ITS^7^ requirements (Figure 3) were not met by the “C” composition, while the maximum IT-CY^28^ (Figure 4) was exceeded after 7 days by the “A” composition. Therefore, mortar “B” was adopted for further tests.

The stiffness modulus at UPC pneumatic press of “B” mortar beams 380 × 63 × 50 mm after 28 days of curing, at the temperature of 10 °C, was E_s_ = 3363 MPa, while in the MTS hydraulic press the obtained value was E_s_ = 4028 MPa. The difference in the results shows that stiffness modulus depends not only on the homogeneity of the materials, but also on the type of equipment and the test method.

**Figure 5:**
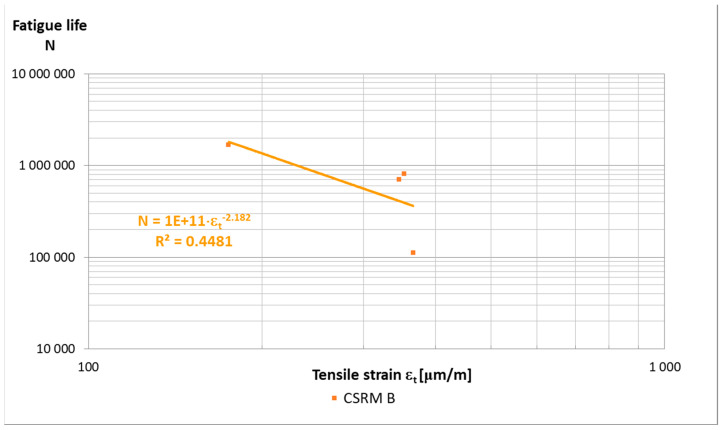
Fatigue life of 380 mm × 63 mm × 50 mm beams made of “B” mortar at 10 °C, after 28 days of curing.

Based on the obtained results (Figure 5), it can be assumed that if tension strain ε_t_ is less than 250 μm/m, the sample will fulfil the assumed criterion of fatigue life.

The analysis of the CSR mortar test results allows to select “B” composition as meeting the technical requirements [15].

## 6. ACCR with the B Mortar Test Results and Analysis

The aim of the research work was to find the optimal composition of the ACCR mixture and to determine the characteristics of its properties in terms of strength, resistance to water and frost, viscoelastic properties, and fatigue life.

**Figure 6:**
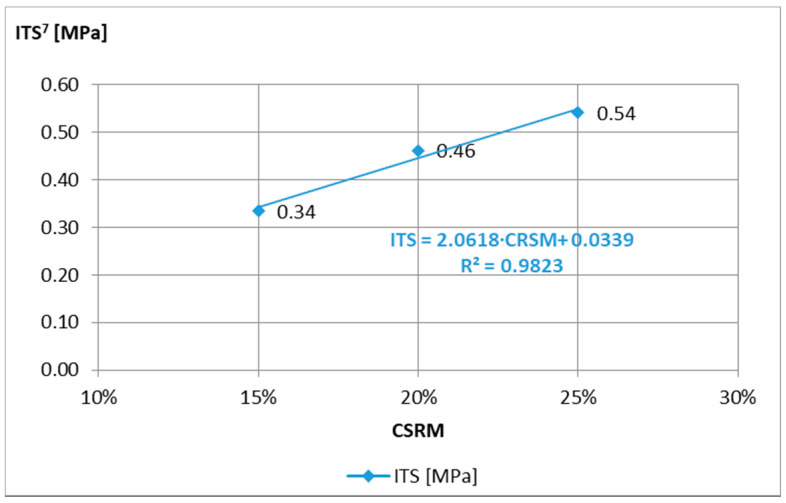
Indirect tensile strength ITS of samples Ø100 mm × 63.5 mm with ACCR depending on the amount of “B” CRS mortar, at the temperature of 5 °C, after 7 days of curing.

Analyzing the results of ACCR tests with various “B” mortar additives (Figure 6), the addition of 15% (m/m) was abandoned due to lower results when the requirements of the indirect tensile strength ITS^7^ > 0.4 MPa.

Water resistance (Table 3) ACCR regardless of the amount of “B” CSR mortar is close to the limit ITSR ≥ 0.7.

**Figure 7:**
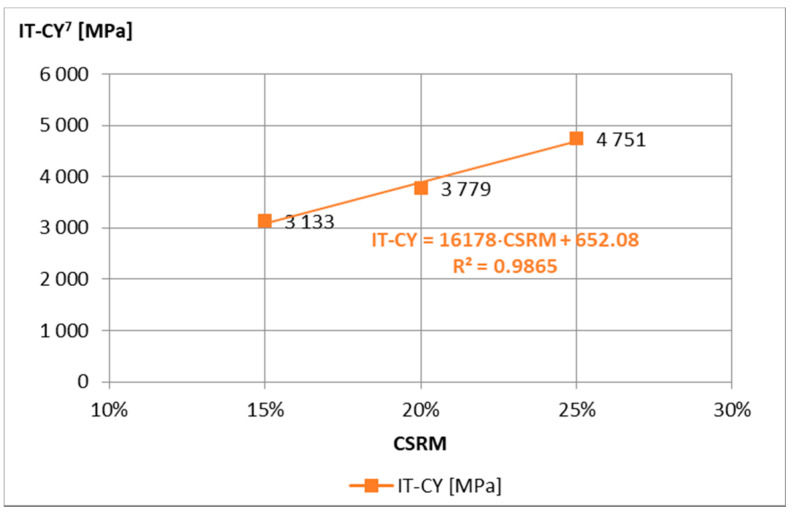
IT-CY stiffness modulus of ACCR with “B” CSR mortar samples Ø100 mm × 63.5 mm depending on the amount mortar, at the temperature of 5 °C, after 7 days of curing.

**Figure 8:**
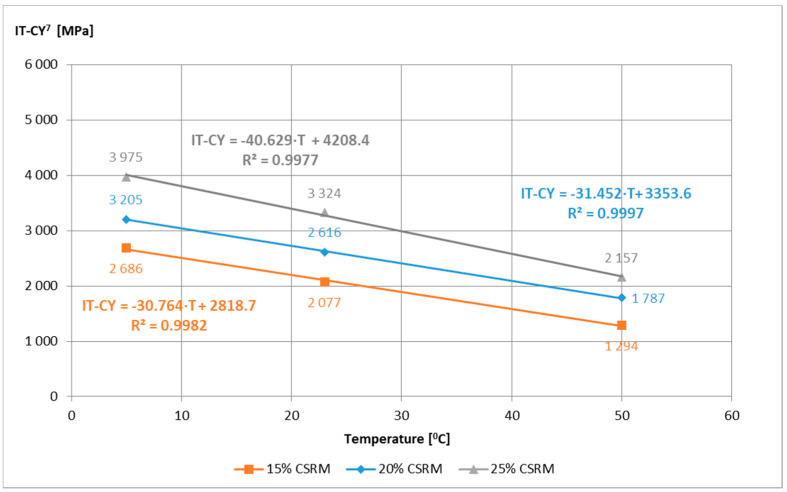
IT-CY stiffness modulus of ACCR with “B” CSR mortar samples Ø100 mm × 63.5 mm depending on the temperature, after 7 days of curing.

**Figure 9:**
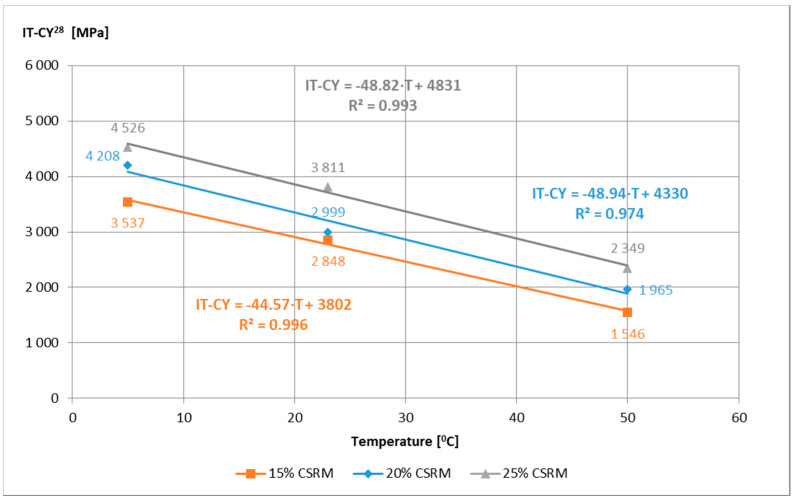
IT-CY stiffness modulus of ACCR with “B” CSR mortar samples Ø100 × 63.5 mm depending on the temperature, after 28 days of maturing.

**Figure 10:**
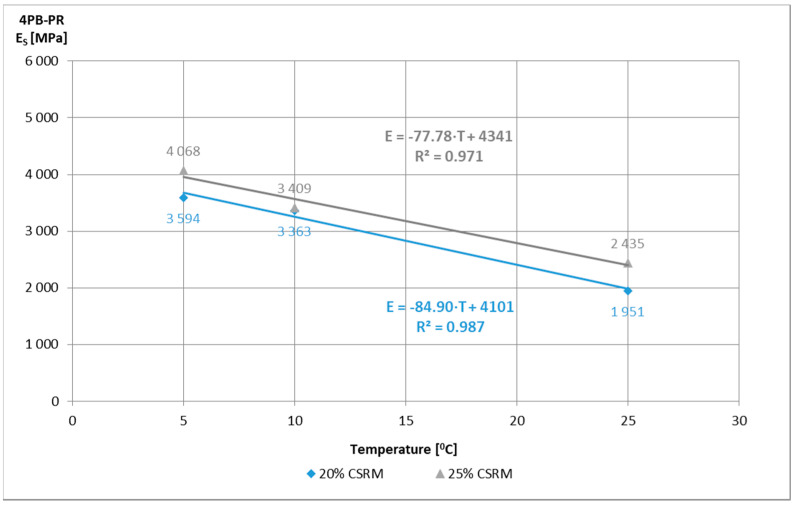
4BP-PR stiffness modulus of ACCR with “B” CSR mortar beams 380 mm × 63 mm × 50 mm depending on the temperature, after 28 days of curing.

Initial tests of IT-CY stiffness modules of ACC with different content of “B” mortar were used to evaluate their values in terms of meeting the requirements according to the technical requirements [15] and to assess their temperature sensitivity. All ACC compositions met the requirements of IT-CY for medium traffic (Figure 7), and their temperature sensitivity is characteristic, i.e., the stiffness modulus at low temperatures is lower than that of asphalt mixes, while at high temperatures they are higher (Figure 8, Figure 9 and Figure 10).

The study of the stiffness modulus with the use of the 4PB test method performed on ACC with the “B” CSR mortar beams in the amount of 20 and 25% at a temperature of 10 °C allowed to determine their average values E_s_^20^ = 5676 MPa and E_s_^25^ = 6303 MPa, respectively. The obtained values are about 50% higher than those tested before (E_s_^20^ = 3363 MPa and E_s_^25^ = 3409 MPa), which may be related to the homogeneity of RAP preparation of samples and the precision of testing in a testing machine.

**Figure 11:**
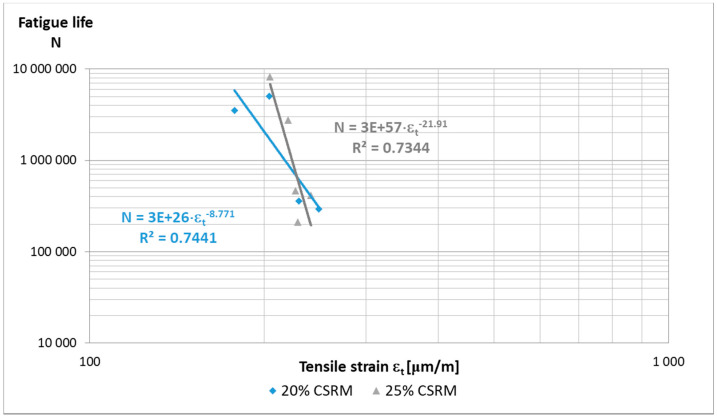
Fatigue life of ACCR with “B” CSR mortar beams 380 mm × 63 mm × 50 mm at 10 °C, after 28 days of curing.

The results of fatigue life tests of ACC with “B” CSR mortar in the amount of 20 and 25% are characterized by high variability (Figure 11), as in the case of fatigue tests, e.g., HMA, where the homogeneity of the samples is decisive. A relatively low value of the correlation coefficient (R^2^ ≈ 0.7) was obtained with the assumed criterion of 50% of the initial value of the stiffness modulus of the tested material. The authors performed the number of cycles to achieve the criterion on only one repetition per strain level and the study was not continued until its destruction. The limit value of the tensile strain in the fatigue test, at which the sample reaches 50% of the initial value of the stiffness modulus after 1 million load cycles, can be estimated at ε_6_ ≤ 200 µm/m regardless of the amount of “B” CSR mortar. The performed fatigue tests should be treated as exploratory due to the small number of samples resulting from the limitations of the research work.

**Figure 12:**
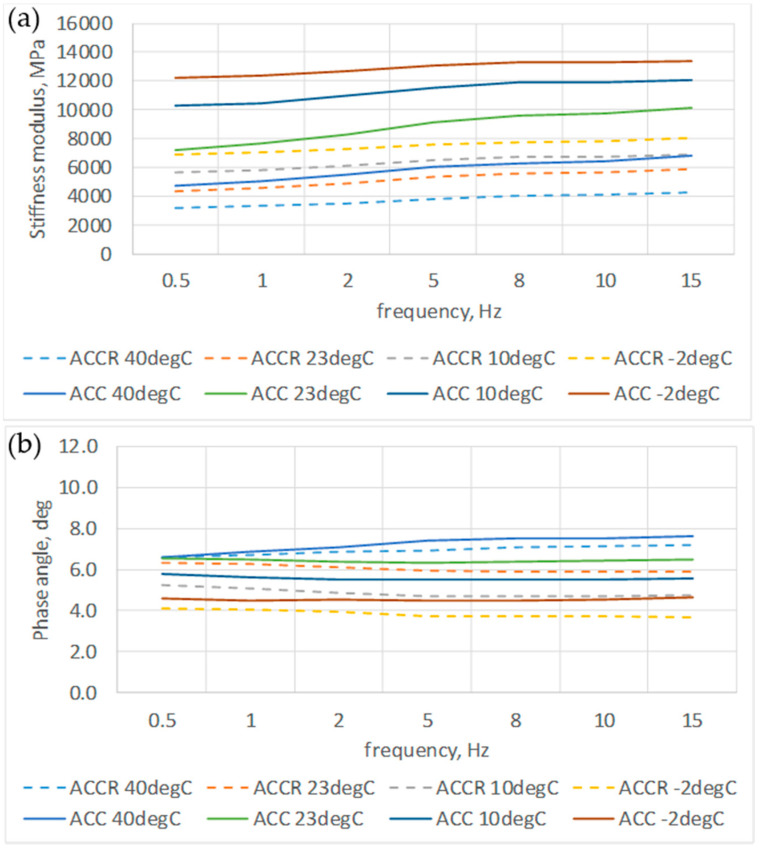
Stiffness modulus E and phase angle vs. frequency for ACC with 20% of “B” CSR mortar and ACC with 20% cement-sand mortar (C:S = 1:1) (**a**) stiffness modulus; (**b**) phase angle φ.

**Figure 13:**
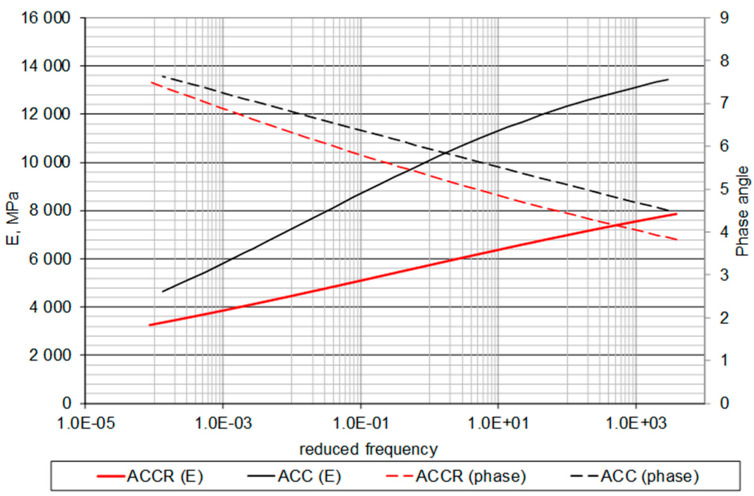
Master curves of stiffness modulus E and phase shift angle φ vs. reduced frequency for ACC with 20% of “B” CSR mortar and ACC with 20% cement-sand mortar (C:S = 1:1).

Complex modulus test results indicate that both ACC and ACCR mixtures are temperature dependent in terms of viscoelastic properties. As the temperature increases, the stiffness decreases and the phase shift angle increases. This is similar behavior to the case of asphalt mixtures (Figure 12). In the same figures it can also be seen that the influence of frequency is much less visible, especially in the case of the phase angle. This shows a different rheological characteristic than in the case of bituminous mixtures. Master curves (Figure 13) indicate that ACC mixture is much stiffer than ACCR. At the same time, it is more sensitive to changing load conditions, which results from the greater slope of the graph.

**Figure 14:**
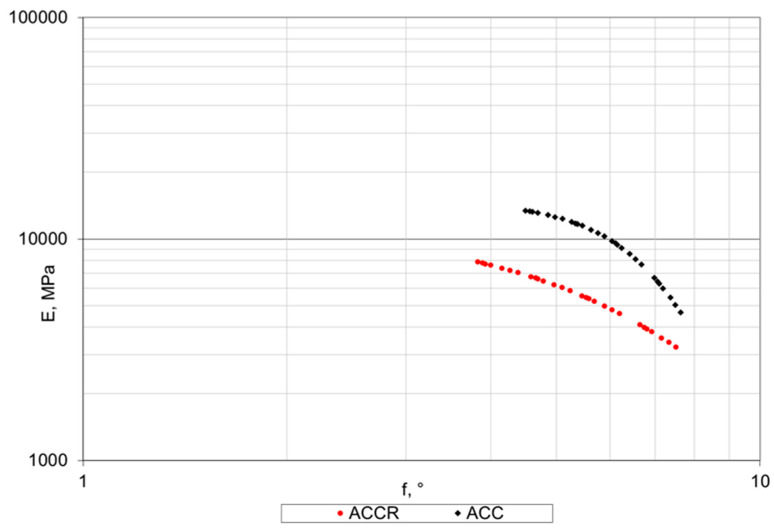
Black plot of stiffness modulus E vs. phase shift angle φ for ACC with 20% of “B” CSR mortar and ACC with 20% cement-sand mortar (C:S = 1:1).

Based on the Black curve (Figure 14), it is possible to evaluate viscoelastic properties of the tested mixtures. A significantly lower value of stiffness of ACCR in relation to ACC can be observed, while the phase shift angle φ is similar and ranges from 4° to 8°. These values indicate that both mixtures have predominantly elastic characteristic and viscous part of modulus is small. In the studies carried out on MCEM [13], the phase shift angle was obtained from 5° to 20°. In case of bituminous mixtures, the phase shift may vary from 5° to 40° degrees.

**Figure 15:**
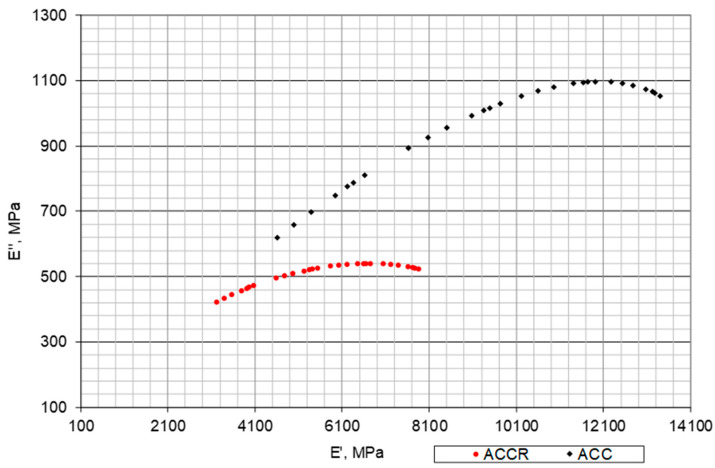
Cole-Cole plot of imaginary modulus E″ vs. elastic modulus E′ for ACC with 20% of “B” CR mortar and ACC with 20% cement-sand mortar (C:S = 1:1).

The Cole-Cole plot (Figure 15) enables the comparison of the real part E‘ and the imaginary E‘‘ of the complex stiffness modulus E *. In the case of ACCR, it can be observed that the imaginary part depends to a small extent on the test temperature and its value is close to 500 MPa, while the real part depends on the temperature, varying in the range of 3000 MPa to 8000 MPa. ACC is much more dependent on temperature and in the case of the imaginary part it ranges from 600 MPa to 1100 MPa, while the real part varies from 4000 MPa to 13,000 MPa.

The determined parameters for Equations (6) and (7) for the analyzed bituminous mixtures are given in Table 4 and Table 5. The R^2^ coefficient of determination indicates a very good relation between the values calculated and the results of the complex modulus tests.

## 7. Conclusions

1. Asphalt-cement concrete with cement-sand-rubber mortar should both fulfil the requirements for indirect tensile strength at temperature 5 °C after 7 days 0.4 MPa < ITS^7^ < 1.0 MPa and the stiffness modulus after 28 days 1500 MPa < IT-CY^28^ < 7000 MPa depending on traffic load.
2. Asphalt-cement concrete with 20% cement-sand-rubber mortar “B” causes circa 50% decrease on the indirect tensile strength and stiffness modulus compared to ACC with 20% sand-cement mortar.
3. Water resistance of asphalt-cement concrete with 20% “B” cement-sand-rubber mortar is low, close to minimum limit ITSR ≥ 0.7.
4. The fatigue tests of asphalt-cement concrete with 20% “B” cement-sand-rubber mortar fulfil requirements for pavement fatigue life, where loss of the initial value of the stiffness modulus after 1 million cycles of load is less than 50% when the tensile strain ε_6_ of the sample is less than 200 µm/m. The tests should be treated as preliminary, they should be continued with a greater number of repetitions and with the possibility of extending the test duration to over 50% of the stiffness reduction.
5. Asphalt-cement concrete with 20% of “B” cement-sand-rubber mortar causes a decrease of temperature and frequency load influence on complex stiffness modulus compared to asphalt-cement concrete without rubber powder addition.
6. The following advantages of the use of ACC with rubber powder are possible:
  - reduction of transverse cracks and weakest points in base course by adding CSR mortar to the RAP,
  - ensuring the fatigue life of the base course by addition rubber powder to cement matrix,
  - increasing the homogeneity of the base course by using mobile concrete batching plants dosing RAP to prepared CSR mortar.

## Figures and Tables

**Table 1:** Grading of constituent materials.

Sieve # [mm]	Passing [%]		
	RAP 0/31.5 mm	Washed Sand 0/2 mm	Rubber Powder 0/1 mm
63	100.0	-	-
31.5	98.7	-	-
16	89.9	-	-
8	68.2	-	-
4	46.2	100.0	-
2	32.2	98.5	100.0
1	23.5	91.5	84.8
0.5	13.8	59.7	37.4
0.125	8.2	1.4	1.0
0.063	7.8	0.4	1.0

**Table 2:** Mortars composition (m/m) [%].

CSRM	Water	Cement 42.5 R	Rubber Powder 0/1 mm	Washed Sand 0/2 mm
A	12	29	18	41
B	12	29	24	35
C	13	29	29	29

**Table 3:** ITSR water resistance of ACCR with B mortar samples Ø100 mm × 63.5 mm, tested at the temperature of 5 °C, after 28 days of curing.

Amount of “B” CSR Mortar in the ACC (m/m) [%]	Dry Samples [MPa]		Soaked Samples [MPa]		ITSR ITS_s_/ITS_d_
	ITS_d_	IT-CY_d_	ITS_s_	IT-CY_s_	
15	0.30	1969	0.20	712	0.68
20	0.39	2308	0.28	930	0.73
25	0.38	2367	0.26	1047	0.69

**Table 4:** Parameters of the stiffness modulus (E) master curve, Equations (5) and (6).

	Mixture	Stiffness Modulus	
Parameter		ACC	ACCR
δH	[J/mol]	98,474	103,604
E_0_	[MPa]	0.010	0.010
$E_{\infty }$	[MPa]	15,239	10,873
c	[-]	0.167	0.103
d	[Hz]	0.511	0.895
R^2^	[-]	0.996	0.993

**Table 5:** Parameters of phase angle master curve θ, Equation (7).

	Mixture	ACC	ACCR
Parameter			
θ_0_	[deg]	44.681	31.878
a	[s]	−0.585	0.000
b	[s]	2.120	5.002
α	[-]	0.015	0.046
β	[-]	0.015	0.046
R^2^	[-]	0.958	0.951

## Data Availability

Data available on request due to restrictions of privacy. The data presented in this study are available on request from the corresponding author. The data are not publicly available due to privacy.
